# Translating knowledge on placebo and nocebo effects into clinical practice

**DOI:** 10.1097/PR9.0000000000001142

**Published:** 2024-03-25

**Authors:** Elif Buse Caliskan, Ulrike Bingel, Angelika Kunkel

**Affiliations:** Department of Neurology, Center for Translational Neuro- and Behavioral Sciences (C-TNBS), University Hospital Essen, University of Duisburg-Essen, Essen, Germany

**Keywords:** Placebo, Nocebo, Nocebo hyperalgesia, Placebo analgesia, Treatment expectation, Expectation

## Abstract

**Introduction:**

Positive and negative treatment expectations are powerful modulators of health and treatment outcomes. A substantial part of treatment success is due to contextual factors modulating patient's expectations towards a treatment. Consequently, treatment expectations should be a target of therapeutic interventions themselves.

**Objectives:**

This article highlights the neurobiological underpinnings of treatment expectations as well as strategies to modulate contextual factors to optimize treatment outcomes in daily clinical settings.

**Methods:**

This clinical update aligns with the 2022 IASP Global Year Translating Pain Knowledge into Practice and selectively reviews the best available evidence and practice.

**Results:**

The effects of treatment expectations, also known as placebo and nocebo effects, are observed in various clinical conditions and physiological systems. However, most of our knowledge comes from the field of pain, where expectation effects substantially contribute to overall analgesic treatment outcomes. Experimental placebo analgesia paradigms provide the best illustration of how analgesic effects can be attributed not only to a pharmacological or specific treatment, but instead are the result of the expectation towards the treatment. The impact of expectations on treatment outcome is highly variable between individuals, and the identification of factors predicting an individual's response has proven to be challenging. Further research is required to provide personalized treatment strategies for the daily clinical practice.

**Conclusion:**

Patient's previous experiences and expectations are powerful modulators of treatment efficacy, tolerability, and adherence. By providing a comprehensive overview of recent advances in this field, this review offers valuable insights for clinicians and researchers seeking to improve patient–clinician interaction.

## Abstract 2

Treatment outcome is strongly influenced by treatment expectations. Modifying expectations by targeting contextual factors can substantially improve therapy success, making them a valuable focus for therapeutic interventions.

**Key Points**

- Placebo and nocebo effects are complex neuropsychological phenomena that are common in all fields of medicine, but particularly strong in the field of pain.
- The key psychological determinant of placebo and nocebo effects is positive and negative treatment expectation.
- The effects of positive and negative treatment expectation, aka placebo and nocebo effects, can substantially modulate treatment efficacy, tolerability, and adherence.
- Placebo effects can enhance the efficacy of a treatment through positive treatment expectations, whereby nocebo effects can induce side effects or abolish the treatment effect through negative treatment expectations.
- Addressing and optimizing patient's expectations as well as previous experiences about a treatment could improve patient's adherence and compliance to a therapy.
- Both effects can be modulated by communication strategies to reduce negative expectations, anxiety, and fear of patients.
- Open-label placebos could provide a unique opportunity to circumvent the deceptive nature of classical placebo treatments by informing the patients about the mechanisms underlying placebo and nocebo effects.

## 1. Introduction

Positive and negative treatment expectations can enhance or diminish the effect of active treatments and influence the frequency of observed side effects and thus the overall success of a treatment. The beneficial effects, which have no causal relationship with the pharmacological action of the administered drug, are commonly defined as placebo effects. By contrast, unfavorable effects are referred to as nocebo effects.^9,27,29,34,48,70^ Recent experimental and clinical studies have demonstrated that placebo and nocebo effects represent complex neuro-psycho-biological phenomena involving the activation of different parts of the central nervous system and peripheral physiological mechanisms. In the case of placebo hypoalgesia and nocebo hyperalgesia, these mechanisms affect the perception of pain and response to analgesic treatments. Expectations can be induced in various ways, including learning processes induced by firsthand experiences or observations, patients' relationships with healthcare providers, and any previous information regarding treatment, such as verbal or written information.^48,70^ The effects of expectations are best exemplified in experimental placebo hypoalgesia paradigms, where analgesic effects cannot be attributed to any pharmacological or other specific treatment but to the expectations towards the treatment.^44,67^ Of importance, positive and negative treatment expectations also modulate the response-active medical treatments, including pharmacotherapy, as is showcased in the so-called open-hidden drug paradigms.^26^

A better understanding of how expectations affect pain and other treatment outcomes is indispensable to the development of systematic interventions in a clinical setting. Thus, promoting placebo and reducing nocebo effects in routine clinical practice has the potential for improving treatment efficacy, tolerability, and compliance. This article highlights the neurobiological underpinnings of placebo and nocebo effects, as well as strategies to modulate contextual factors and optimize treatment outcomes of analgesic treatments in daily clinical settings by addressing the following questions: How can positive treatment expectations be targeted to optimize treatment efficacy, and how can the detrimental effects of negative treatment expectations be mitigated to optimize tolerability and adherence to analgesic treatments?

## 2. Psycho-neuro-biological correlates and mechanisms

**Figure 1.**
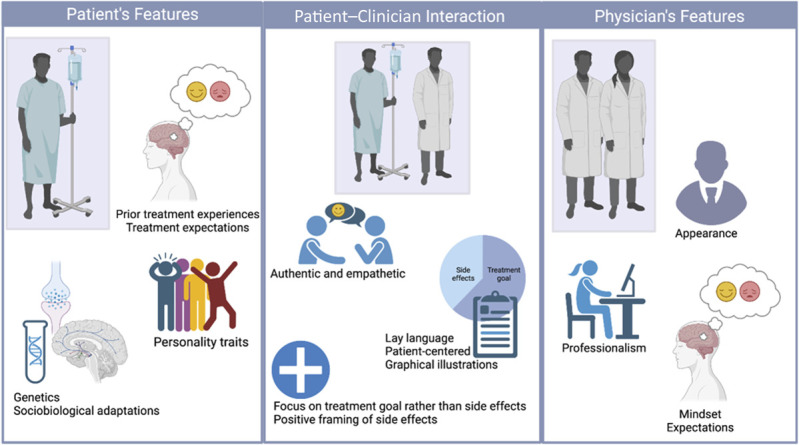
Patient-related and physician-related factors that contribute to placebo and nocebo effects.

From a psychological perspective, placebo and nocebo effects are mediated by several factors associated with expectations of treatment outcomes (Fig. 1). These expectations are acquired through various ways, including firsthand experiences of medication effects and side effects (eg, nausea after chemotherapy or analgesia after taking pain medication), instructions and information provided by healthcare professionals (eg, informed patient consent), and social observation (eg, directly observing symptom relief in another person undergoing the same treatment).^21^ Positive and negative expectation effects can be found in different clinical conditions and physiological systems, but most of our knowledge comes from the field of pain management.^67^ Placebo hypoalgesia refers to the relief of experimentally induced pain following the administration of an inert treatment and the expectation that a potent analgesic substance is being administered. By contrast, nocebo hyperalgesia involves the exacerbation of pain due to negative expectations and beliefs about pain.

But also the effects of an analgesic treatment can be modulated by expectations,^19^ which revealed the analgesic effect of remifentanil, a potent μ-opioid agonist, on experimentally induced heat pain in healthy participants. The administration of the drug was combined with 3 different verbal instructions and previous experiences (induced by a conditioning session). Of interest, the positive expectancy of analgesia doubled the analgesic effect of the study drug, while the negative expectancy almost nullified it. The authors also examined brain activity during the experiment and observed increased neural activity in the brain regions mediating mood and anxiety such as the hippocampus and medial prefrontal cortex when analgesia was impaired due to negative expectations. By contrast, not only parts of the descending pain modulatory system such as the anterior cingulate cortex but also the striatum were associated with expectation-augmented analgesia.^19^ The descending pain modulatory system can modulate the transmission of nociceptive information in the spinal cord through descending signals from the supraspinal structures. This system acts as a regulatory pathway, providing bidirectional central regulation of nociception, which is essential for the organism's survival.^18^

A modulatory role of expectations for treatment outcomes has also been demonstrated in multiple pain treatment modalities, such as migraine treatment,^3,61^ acupuncture,^57,82^ multimodal pain therapy,^58^ pulmonary rehabilitation,^36^ and to increase pain endurance in sport competitions.^16^

Expectations are induced not only by verbal instructions but also by the awareness of being administered a drug, which can significantly increase its analgesic effect.^54^ Open-hidden paradigms for drug administration provide the most compelling illustrations of placebo effects in routine clinical practice. In these paradigms, a drug can be administered in 2 distinct ways: open administration, where the patient sees the drug being administered by a health care professional, and hidden administration, where an automated machine administers the drug without the patient's knowledge. Open administration mirrors the standard clinical practice, whereas hidden administration isolates the drug's true pharmacological effects from the psychosocial context and its influence. Therefore, hidden administration can attenuate the effectiveness of treatment. After comparing the open and hidden applications of 5 common painkillers (morphine, buprenorphine, tramadol, ketorolac, and metamizole), Colloca et al.^26^ found that the dose required to achieve the same analgesic effect for all drugs was much higher. In addition, the pain intensities were rated higher following the hidden administration compared with the open administration of the drug. These findings were replicated in patients with postoperative pain, where a 50% larger dose was needed to reduce the pain^2^ and in the treatment of anxiety and deep brain stimulation of Parkinson disease.^15,53^ These findings underline the pivotal role of expectations in treatment efficacy and outcomes.

Explicit information about the side effects can also affect outcome expectancy or lead to their occurrence, also known as the “self-fulfilling prophecy.”^41^ Participants rated a nonpainful tactile stimulus as painful and a low-intensity pain stimulus as high-intensity pain after being administered a physiological saline solution with verbal instruction that the drug would worsen the pain.^10^ Furthermore, patients in the placebo arm of double-blind clinical trials often show similar response rates^43^ and side effects^68^ compared with the patients undergoing active treatment. In a study examining sexual dysfunction under finasteride therapy, 43.6% of the men who were informed about the possible “occurrence of erectile dysfunction, decreased libido, and ejaculation disorders” reported these side effects. By contrast, only 15.3% of the patients not informed about these potential side effects reported them.^62^ In another study on the beta-blocker atenolol for cardiac disease and hypertension, 31% of the patients who were specifically explained about sexual side effects and erectile dysfunction reported these potential side effects in contrast to 16% of those who were not informed about these side effects.^73^ In a systematic review, Amanzio et al.^1^ illustrated that the adverse events experienced in placebo arms of clinical trials correlate with the adverse events expected of the experimental compound. Anticonvulsant placebos, for example, caused more anticonvulsant-specific side effects, such as anorexia, fatigue, and memory difficulties, than placebos used in nonsteroidal anti-inflammatory drug or triptan trials.

Furthermore, many symptoms such as pain,^10^ nausea,^52^ shortness of breath,^32^ and itchiness^12^ have been reported to be triggered or intensified through negative treatment expectations. Weissenfeld et al.^78^ reported an increased rate of additional adverse events after generic substitution of a medication because of nocebo effects, leading patients to become nonadherent and consequently discontinue appropriate therapy. In this case, a detailed explanation from the physician to alleviate patients' fears by assuring them that the pharmacologically active substance is identical was shown to improve adherence to the generic substances.

Associative learning processes are suggested to be involved in the placebo and nocebo effects. For instance, during classical conditioning, a neutral cue that has previously been associated several times with either positive or negative effects of medication later triggers these effects even without the administration of the drug.^74^ Similar relapse rates were observed between patients who underwent a reduction of the glucocorticoid dose through intervals with a placebo (a dose-extending placebo) and patients taking the full glucocorticoid dose.^25^ The mere observation of an allergen sealed in a container can induce asthma attacks in patients with asthma.^32^ Women receiving chemotherapy for breast cancer observe anticipatory nausea when they encounter a previously neutral contextual stimulus associated with infusions, such as hospital smell.^45^ These learning mechanisms are not limited to firsthand experiences; observing others undergoing treatment can induce placebo and nocebo effects. This phenomenon of induced effects was demonstrated in a study in which only 1 group member was informed about headaches triggered by hypoxia before the group experienced hypobaric conditions in the mountains.^14^ The informed participant later “infected” other members of the group, depending on how much social contact they had with that person.^9^ In addition to direct personal contacts, information disseminated by social media and the press can also induce placebo and nocebo effects. A recent meta-analysis of 20 studies showed high placebo response rates in the placebo arm of cannabinoid clinical trials, leading to a limited superiority of cannabinoids compared with placebo because of the high attention of media with a strong positive bias.^37^ Nocebo responses can also be spread through lay media. After myocarditis was discussed as an adverse event after the Pfizer-COVID-19 vaccine by the media in New Zealand, the reports of chest discomfort increased by 190% following the first media coverage of vaccine-related adverse events.^56^ Negative media coverage of statins has also been linked to higher reports of statin-related side effects.^59^

Neurobiological underpinnings of placebo hypoalgesia have been investigated in multiple pharmacological and neuroimaging studies in recent years. The neurobiological mechanisms underlying these effects consist of various neurotransmitter and neuromodulator pathways, including the involvement of endogenous opioids,^33^ endocannabinoids,^11^ dopamine,^55^ serotonin, endorphins, oxytocin,^50^ and vasopressin.^28^ The orchestration of these neurohormonal responses is believed to be facilitated by the autonomic nervous system, which establishes a physiological link through a combination of sympathetic and parasympathetic activation. This connection may bridge the gap between these neurohormonal responses and the organ-specific reactions observed in the periphery.^60^ However, the distinct interaction of central nervous system and peripheral physiological mechanisms underlying placebo analgesia/hyperalgesia and other placebo/nocebo effects is only poorly understood.

In neuroimaging studies, placebo hypoalgesia has been reported to be linked to decreased activation in pain-responsive regions, such as the rostral anterior cingulate cortex, insula, and thalamus, and increased activation of pain modulatory regions, such as the dorsolateral prefrontal cortex (DLPFC), orbitofrontal cortex, and the periaqueductal grey. The DLPFC seems to play a crucial role in initiating and maintaining expectancy effects on pain.^33,77^

This role of DLPFC in expectation effects on pain is further corroborated by findings in Alzheimer disease, in which placebo hypoalgesia was attenuated with progressive degeneration of the prefrontal and anterior cingulate cortices.^13,31^ The nocebo effect, on the contrary, has been shown to be linked to the secretion of the anxiogenic neuropeptide cholecystokinin. The exacerbation of experimentally induced pain through verbal instructions was blocked by proglumide, a cholecystokinin receptor A and B antagonist. This type of nocebo hyperalgesia has been reported to be associated with increased activity of the hypothalamic-pituitary-adrenal axis, which was reduced by benzodiazepine diazepam,^12^ suggesting the role of anxiety in nocebo effects. Increased activation of the afferent pain circuitry, including the spinal cord, has been demonstrated.^27,34^ Whether the modulatory pathways initiating nocebo hyperalgesia overlap with those initiating placebo hypoalgesia is not yet fully understood. A few neuroimaging studies that have investigated nocebo hyperalgesia point towards a special role of the hippocampus; however, these findings warrant further investigation.^20^

The impact of expectations on treatment success varies considerably between individuals. Identifying a common psychological mechanism that would differentiate a placebo responder from a nonresponder has been challenging because these responses consist of biologically diverse phenomena [NO_PRINTED_FORM]. Existing research supports the notion that more anxious patients,^75^ whose medical history encompasses unexplained symptoms^6^ or who are under greater psychological distress,^30^ are more prone to develop nocebo responses. By contrast, optimism,^38^ an individual's functional and structural properties of brain connectivity, so-called resting state connectivity^71^ and genetic traits in dopaminergic,^39^ opioidergic,^65^ and endocannabinoid^66^ have been associated with placebo hypoalgesia. For more detailed information on placebome, we refer the interested reader to Ted Kaptchuk's review on genetic influences in the placebo effect.^40^

Although identifying patients most likely to develop placebo and nocebo responses would be advantageous, there is no consistent evidence in larger samples of such predictors. Concerted efforts, including international cooperation, which pool large datasets from experimental and clinical studies will hopefully provide a clearer picture in the future to inform personalized treatment strategies.

## 3. Clinical implications

### 3.1. Using placebos in an ethically acceptable way

The traditional (ie, deceptive) application of placebos in the clinical context is limited by ethical constraints because they compromise patients' autonomy.^42^ Clinicians are usually aware of the classical definition of placebos, which is the administration of “inert substances” without any specific effect on a patient's condition. Although this type of placebo application is regarded as unethical, surveys indicate that between 17% and 80% of doctors have applied “pure” placebos at some point in their professional career.^35^ Even more frequently, they prescribe active drugs without any indication, so-called “impure placebos,” eg, prescribing antibiotics in case of a viral upper respiratory infection. Although not directly considered a placebo, clinicians seem to accept this clinical practice as a placebo intervention when asked explicitly.

Novel strategies of so-called open-label placebo (OLP) treatments circumvent this ethical dilemma by informing the patient about the nature of the placebo treatment before administering it. In this context, a patient-oriented presentation of OLPs seems to be helpful to elicit placebo responses.^47^ Besides clearly stating that OLPs are inert substances containing no pharmacologically active compounds, most previous studies have emphasized the “powerful nature of placebo effects” and potential ways of action—such as conditioned bodily responses.^80^ There is promising evidence for the beneficial effects of OLP treatments on experimental, acute, and various chronic pain disorders^80^ (such as migraine,^46^ chronic back pain,^23,51^ and irritable bowel syndrome^47^), as well as on other subjective complaints, such as cancer-related fatigue^83^ or depressive symptoms.^23,49,55^ OLPs also improved adherence to methadone maintenance treatment for patients with opioid use disorders. Open-label placebos were shown to improve the 90-day retention rates and sleep quality of participants compared with the treatment as usual.^8^

Nevertheless, specific patient-level predictors of the response to OLP remain unclear. In a study conducted by Ballou et al. on patients with irritable bowel syndrome, the response to OLP treatment was negatively correlated with pain catastrophizing (PC) and positively with visceral sensitivity (VS), which measures different aspects of symptom-related anxiety and distress. Pain catastrophizing reflects rigid thinking and a sense of powerlessness in coping with pain, whereas VS interferes with self-efficacy as the ability to exert an impact on or effectively manage one's own symptoms. In the light of these results, the authors discussed that flexible thinking could predict the improvement in OLP response, whereas a sense of helplessness might hinder it. The underlying message of OLP treatments, which is paradoxical (“this inert pill may help”) and emphasize the body's self-healing ability, may necessitate cognitive flexibility.^4^ On the contrary, studies administering OLPs as dose extenders^7,69^ rely predominantly on placebo responses through classical conditioning. Exploring the differences between these approaches could be a compelling topic for future applications of OLPs in the clinical context. Various concepts have been discussed to underly or contribute to the beneficial effects of OLP ranging from the “Bayesian brain,”^64^ resolving cognitive dissonance,^4^ or conditioned responses.^7,69^ Of interest, also the role of expectations, the key mechanisms underlying deceptive placebo treatments, is unclear to date. Thus, future studies must explore OLPs mechanisms of action, longer-term effects, and patients who may particularly benefit from them.

### 3.2. Harnessing placebo effects rather than the placebos to augment analgesic treatment outcomes

Exploiting placebo effects does not rely on the use of placebos. As the key determinant of placebo effects, treatment expectation can and should represent a therapeutic target. So how can treatment expectations be used systematically in the clinical setting? As showcased in the open-hidden paradigms, a substantial part of the overall treatment success can be attributed to contextual factors that modulate patients' treatment expectations. Physicians should be aware of how to modify expectations to improve treatment outcomes. In this study, we summarize different evidence-based approaches to apply these effects in clinical practice and capitalize on contextual factors' known role in health care.

#### 3.2.1. Verbal information

Patient–clinician interaction is pivotal in generating placebo and nocebo responses by affecting patients' expectations and beliefs.^22^ Healthcare professionals should understand how they can unwittingly trigger nocebo responses. They should be aware of the communication strategies they can use to maximize placebo effects and minimize nocebo effects (Table 1). Empathetic and authentic communication with the patients, explaining their illness and the planned treatment in an easy-to-understand manner with lay terms and taking time to answer patients' questions thoroughly are known to help avoid misunderstandings, prevent side effects, and improve treatment compliance.^11^ The patients' education level and preexisting knowledge should also be determined to provide adequate information on diagnosis, illness, treatment, and its aims and possible adverse effects. Considering patients' expectations and previous (failed) treatment experiences could also reduce nocebo effects.^51^

**Table 1:** Strategies for enhancing clinical interventions, adapted from Bingel et al.^17^

Expectation and learning-based strategies	Patient–clinician interaction
Assess patient's expectations regarding the intervention and side effects Determine previous (failed) treatment experiences Focus on desired treatment outcome before explaining potential risks Provide strategies to cope with side effects Use visual tools, such as videoclips, or information leaflets with graphical presentation of information and lay language Deliver evidence-based information on the planned treatment and the illness instead of anxiety-inducing unproven sources Choose pretreatment with lower side effect profile	Communicate in an authentic and empathic manner Assess patients' level of knowledge Give adequate information on disease, prognosis, treatment, aim of the treatment, and possible side effects Ask proactive questions to confirm patients' understanding of treatment goal and side effects (eg, let the patient summarize) to prevent maladaptive expectations and misunderstandings Take time to listen and answer to the concerns and questions of the patients to promote compliance

Informed consent promotes patients' right to be informed and enables them to make autonomous decisions regarding the implementation of the therapeutic measures in question. However, informed consent also requires a detailed explanation of side effects, which can produce a poorer response to the treatment through nocebo effects.^79^ Therefore, communication strategies to reduce patients' negative expectations, anxiety, and fear while maintaining informed consent are critical.^17^ This would be partly possible by strategically combining information about potential side effects with the desired therapeutic effect, improving patients' condition. The clinician can use the “primacy” or “recency” effects here. These effects describe a person's ability to remember the first and the last topic mentioned in a speech. Thus, it would be beneficial to emphasize the desired effects of the therapeutic intervention at the beginning and end of communication with the patient instead of focusing on possible side effects. Accordingly, these side effects should be mentioned in between.

Another potentially helpful strategy is the positive framing of side effects, which modulates how the brain processes given information.^5^ Should the possibility of *not* experiencing side effects (positive outcomes) be highlighted against the possibility of experiencing side effects (negative outcomes), patients' expectations of side effects may be reduced. For example, before the administration of influenza vaccination, positively framed information on the percentage of people not experiencing vaccine-related side effects resulted in a reduction in the reporting of side effects and loss of working hours, when compared with informing patients about the probability of side effects.^63^ In a study conducted by Wilhelm et al.,^81^ participants who were informed that the side effect of dizziness is “a sign that drug had started to work” (positive framing) rated the intensity of side effect less than the participants who were explained that dizziness is “an unpleasant but well-known side effect” (neutral framing). In addition to explaining side effects, the clinician should inform the patient about potential coping strategies in case of transient adverse effects to promote perceived self-efficacy and treatment adherence and compliance.^41^

Especially in situations perceived as life-threatening, such as surgeries, accidents, or acute severe diseases, patients are in a psychological state that leads to an altered perception of time, space, and consciousness, are more receptive to negative suggestions, and have a restricted awareness of their surroundings, which may be described as a natural trance state.^24^ They are particularly vulnerable to misunderstandings through medical terminology (“We looked for metastases—the result was negative.”), ambiguities (“We're putting you to sleep now, it'll soon be all over”), phrasings causing uncertainty (“This medication may help”), or emphasizing the negative (“You are a high-risk patient”). In addition, the use of negative words such as “burn,” “sting,” “bad,” or very simply “pain” can trigger anxiety and thus facilitate pain perception.^41^ It would be desirable to focus on the benefits of the treatment rather than its risks. The intensity of pain caused by the administration of a local anesthetic injection was reduced by the positive verbal explanation, “We are going to give you a local anesthetic that will numb the area, and you will be comfortable during the procedure,” as opposed to the negative verbal explanation “You are going to feel a big bee sting; this is the worst part of the procedure.”^76^

#### 3.2.2. Previous treatment experiences

Given that often, no effective therapy is available to induce a positive treatment experience, especially in chronic pain conditions, physicians and healthcare professionals should be aware of the detrimental effects of previous negative experiences, which can be generalized over time, and various treatment approaches.^50,84^ If there is a choice, therapies with a low side effect profile should be chosen as initial therapies to prevent negative associations that could affect the patients' adherence and compliance with subsequent therapy attempts. Therefore, considering patients' experiences and preferences regarding analgesic treatment should be integrated into the treatment plan. Fear and anxiety of patients can be soothed with the help of improved patient information systems, eg, through illustrative methods, such as video clips of other patients reporting about their positive treatment experiences and their coping strategies against unwanted adverse effects, and through information leaflets designed to minimize negative expectations regarding side effects using a patient-oriented language. Enhancing positive treatment expectations through strategies involving social observational learning is another promising avenue for future research.^72^

## 4. Concluding remarks

In conclusion, treatment expectations and the resultant placebo and nocebo effects substantially modulate the efficacy, tolerability, and adherence to analgesic treatments. Consequently, treatment expectations should be a target of therapeutic interventions itself. Treatment expectations are malleable and can be systematically changed in an ethically acceptable manner to maintain patients' autonomy and foster self-efficacy. Such strategies include optimizing the information that patients receive regarding their treatments, acknowledging and considering contextual factors in treatments and treatment settings and optimizing and individually tailoring communication strategies between patients and healthcare providers. These approaches promise to promote positive treatment expectations that lead to placebo effects and avoid negative treatment expectations, which could optimize treatment efficacy and adherence while reducing treatment discontinuation rates.

## Disclosures

The authors have no conflict of interest to declare.
